# Letter from Our Own Correspondent in Paris, Dr. Weymann

**Published:** 1887-11

**Authors:** 


### FOREIGN CORRESPONDENCE.

NEWSY LETTER FROM OUR SPECIAL CORRESPONDENT.

PARIS, FRANCE, October 31, 1887.

*Editor Daniel's Texas Medical Journal.*

DEAR SIR: In opening this correspondence, I hope that not only will your readers appreciate your enterprising spirit, but also that I may be able to satisfy them with my attempts to give them short and practical accounts of the progress in medicine both in the European institutions and among the profession at large.

If one remembers how incomplete the influence of any of the usual methods of medication is in catarrhal jaundice, it will be welcome news to hear of a treatment which is said to effect a cure with almost mathematical surety. True, the plan is not entirely new, for it was practiced some time ago by several prominent German physicians, but for some reason has failed to come to general notice. All the advocates of this treatment are full of its praise, and even under the rigorous trials and observations of a French hospital, it has justified all anticipations. The average duration for a cure is from six to eight days, although frequently the second day may have wrought a decided improvement. If one notices carefully the color of the stools, and tests carefully and systematically for the bile pigments in the urine, it is possible to register the cure with almost statistical exactitude. Before detailing the treatment, I will add that a very complete table of successes is, given in the report, and that no failure was mentioned in all the cases of jaundice ot catarrhal origin.

Besides the general care, the patient is to have an enema or clyster every day. The originators of the plan employed it in the evening only, but in France the injection is given in two divided doses, in the morning and evening respectively. No medicine besides the agency of cold is employed. One pint of water of about the temperature of 50° F. is used the first two times. From day to day the temperature is increased from two to four degrees, until at 70° F. no further increase is advisable. The enemata are to be taken in bed, and, although the beginning injection is very cold, no chilly sensations or other unpleasant feelings are usually complained of.

As a first class cardiac stimulant for cases of valvular insufficiency, strophantine has been talked about. Especially Vienna seems to be highly pleased with it. It is said to be by far superior to digitalis, and, in cases of dropsy, it eliminates the transudation with great rapidity. It is of course powerless when the insufficiency is multiple and the period of compensation has passed already. Stimulating the heart muscle to increased activity, as it does, it is of little use when the cardiac walls are hypertrophied to such an extent as to need no increase in force. In all those cases, the lesions cannot be overcome, or even palliated, on account of their enormous extent. When administered, the tincture may be combined with digitalis. Its main advantage rests in the fact that strophantine may be given hypodermically in doses of about 1-64g; the tincture is from gttx-xxx.

A very interesting subject was discussed at the last meeting of the Academy of Medicine. It was the article of Dr. Verneuil, on "Imaginary Ulceration of the Tongue." In it he describes a peculiar subjective idea of certain patients who complain of a feeling of soreness of the tongue. They imagine to be able to recognize from their censory impressions the presence of an ulcerating sore; they even will insist, in spite of the assurances of the physician that there is nothing the matter. Cases of this class had come under the observation of nearly all the members, and in making out the clinical import of the peculiar affection, the great bulk of all the evidence went to show some coming nervous trouble. Most frequently it was the precursor of general paralysis. It also was noticed in old cases of syphilis, where its occurrence was attributed to the long use of mercury; for, after leaving out those preparations, an invigorating tonic treatment would soon see the complaint disappear. In those cases where nervous troubles were to be anticipated, treatment was very unsatisfactory, arsenic and alkalis doing, perhaps, the most good. One point was particularly dwelt upon, the happy choice of the name, "Imaginary ulceration," since the appellation of glossodynia, as preferred by some, did not at all apply to such a distinct nervous trouble.

A very nice mode of using cocaine was reported in connection with lithotrity. In patients with heart disease, with kidney troubles and of great age, where the anæsthetic, as well as the healing process, might constitute unpleasant factors, cocaine would perfectly suffice for rendering the natural passages insensible. After the crushing of the stones also the passage of the fragments would cause no pain. Besides, an enormous dilation of the urethral canal would be possible. Usually the work might be done in some three to five sessions, of which none ought to last for a very long time. Concerning the toxic effect of cocaine, there should be no cause for fear. The walls of the bladder were known to be non-absorbing surfaces (?) and it was only after the desquamation of the epithelium that any danger was to be feared. Moreover, by thoroughly examining the urine macroscopically and microscopically, our chances could greatly be foretold. Pus, blood, numerous epithelia, and easily produced hemorrhages, would indicate absorbing surfaces. Even then injections would be safe, provided they were made by gradually increasing the strength of the solution until anæsthesia were complete. The gentleman in question invariably commenced with a solution of one part of the salt to forty of water, and his success never stayed out after coming to a strength of 1:12.

In closing this letter, I will mention the very remarkable discovery of Drs. Spillman & Hanshalter. Those two gentlemen, noticing a great many flies on a consumptive patient in the hospital, were eager to find out whether these insects could be the carriers of the bacillus. They were successful in finding the microbe, not only in the alimentary tracts, but also in the excrementitious matter as taken from the window panes. Their conclusion is to the effect that flies are very potent disseminators of the bacillus, and they have now undertaken a number of inoculations on animals to see what importance this invention might have in a practical respect. The method of examination, by the way, is easy enough for anybody who understands the use of the microscope. As staining agent, Fraenkel's double coloration was employed.

